# Evaluation of a Fully Automated Assay for Detection of Antidengue IgM Antibodies in a Nonendemic Area

**DOI:** 10.1155/jotm/4163150

**Published:** 2025-08-01

**Authors:** Aurélie Guigon, Pauline Coulon, Laura Pezzi, Alexandre Regueme, Cyril Debuysschere, Mouna Lazrek, Didier Hober, Enagnon Kazali Alidjinou

**Affiliations:** ^1^Laboratory of Virology ULR 3610, Lille University, University Hospital of Lille, Lille, France; ^2^Emerging Viruses Unit, Aix-Marseille Univ, Università di Corsica, IRD 190, Inserm 1207, IRBA, Marseille, France; ^3^National Reference Center for Arboviruses, Inserm-IRBA, Marseille, France

**Keywords:** automated assay, dengue, diagnosis, IgM antibodies, RT-PCR

## Abstract

Current French guidelines on the diagnosis of dengue infection recommend both nucleic acid testing and serology as tools for laboratory confirmation. This study aimed to evaluate the performance of the fully automated Virclia IgM assay for the diagnosis of dengue infection. Samples from patients with a suspicion of dengue were prospectively tested using the Virclia Dengue IgM assay (Vircell) and subsequently underwent additional investigations (dengue RT-PCR and conventional dengue IgM EIA) at the French Reference Center for Arboviruses. A total of 104 patients were included with a median age of 34.3 years old and a median time since symptom (TSS) of 6 days. Dengue RT-PCR was positive in 57 patients (54.8%). The agreement was excellent (90.5%; *κ* = 0.81) between RT-PCR and Virclia Dengue IgM assay on samples collected from Day 5 postsymptom onset. On these samples, the sensitivity and specificity of the Virclia IgM assay were 95.7% (95% CI: 84.7%–96.9%) and 96.4% (95% CI: 80.8%–100%), respectively. In addition, the agreement was also excellent between the Virclia Dengue IgM assay and the Euroimmun plate-based Dengue IgM ELISA (92.7%; *κ* = 0.85). In conclusion, the Virclia Dengue IgM assay showed a good performance in the diagnosis of dengue infection and can be recommended in addition to nucleic acid testing to broaden the diagnostic window. The automation coupled with the monotest format is well-adapted for nonendemic areas.

## 1. Introduction

Dengue is the most rapidly spreading mosquito-borne viral disease and is caused by one of the four dengue virus (DENV) serotypes (DENV-1 to DENV-4). DENV are transmitted notably by *Aedes aegypti* (in tropical regions) and *Aedes albopictus* (in temperate regions) [1, 2]. The global burden of dengue is estimated at around 400 million infections per year, 96 million annual symptomatic infections, spreading mainly in Asia (70%), Africa (16%), and the Americas (14%), where dengue disease is a major public health concern [2–4]. The incidence has also recently increased in Europe [1, 5]. In France, dengue fever is considered endemic only in French overseas territories and departments (FODT). Most cases in mainland France are imported from FODT or other areas in America, Asia, or Africa; however, indigenous cases have been described in mainland France, with an increasing number each year. In 2022, 65 indigenous cases were thus recorded, compared to 48 over the previous decade. In addition, 71 mainland departments are currently considered colonized by *Aedes albopictus* compared to 34 in 2016 [6].

Clinical features of dengue infection are variable ranging from mild to severe [1, 3], and symptoms are nonspecific in the early phase. It is thus challenging to differentiate it from the other causes of acute febrile illness, including co-circulating arboviruses. Therefore, laboratory diagnosis is essential to differentiate dengue from other diseases with similar clinical presentation and to implement prevention measures [3, 7].

During the acute phase (0–7 days after symptom onset), current guidelines recommend that laboratory diagnosis should be made by using either one of these test combinations: (i) the detection of circulating DENV RNA by RT-PCR and an IgM antibody test, or (ii) a NS1 antigen test and an IgM detection test [8]. The NS1 antigen is of limited interest in areas with a low incidence such as mainland France. Nucleic acid testing is considered as the gold standard but is not usually available at the local laboratory, or not on a daily basis, even in referral hospitals. A rapid IgM testing can thus be a pivotal first-line screening tool in clinical routine, and is recommended from 5 days after symptom onset, in the French algorithm [6]. In addition, IgM antibodies (Ab) can be detected up to 3 months or longer after infection and then represent a reliable diagnostic marker beyond the acute phase.

IgM Ab can be detected using an enzyme immunoassay (EIA) or an immunochromatographic rapid diagnostic test (RDT). RDTs have been widely implemented especially in endemic settings because they are cost-effective and easy to perform with results usually available in less than 30 min and provide the opportunity for point-of-care diagnosis [8, 9]. In comparative studies, EIAs are more sensitive than RDTs and are usually considered as the gold standard in dengue Ab testing [10, 11].

However, conventional microplate EIAs are less convenient for routine clinical practice due to the turnaround time and the need to test a batch of samples. Automated platforms enable rapid diagnosis through random access testing and are also suitable for high-throughput laboratories. DENV IgM Ab testing is only available on very few fully automated platforms including VIDAS (bioMérieux, France) [12, 13] and Virclia (Vircell, Spain). We here provide the first independent evaluation of the automated Virclia Dengue IgM assay, as compared to results provided by the French Reference Center for Arboviruses (FRCA).

## 2. Materials and Methods

### 2.1. Study Design

**Figure 1:**
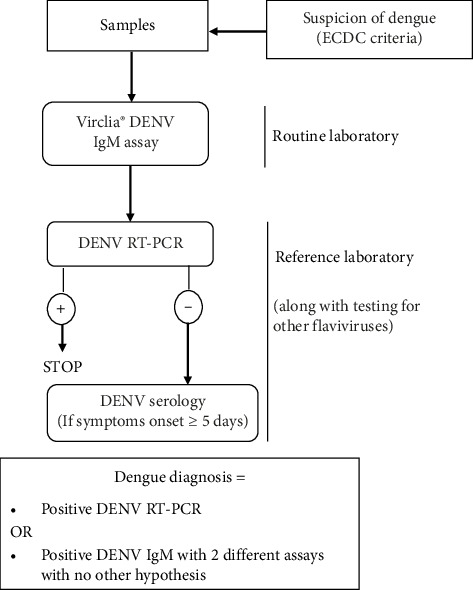
Diagnostic algorithm during the study period. DENV: dengue virus; ECDC: European Center for Disease Control; +: positive; −: negative.

This monocentric evaluation included patients with suspicion of dengue, as defined by the European Center for Disease Control [14], who underwent routine IgM testing in our laboratory, with additional investigations by the FRCA. Serum samples were collected and prospectively tested between 2020 and 2024. The diagnostic algorithm used for the study is shown in Figure 1.

### 2.2. Ethical Aspects

Patient records were anonymized and de-identified prior to analysis. This study was performed in strict compliance with the French reference methodology MR‐004 established by the French National Commission on Informatics and Liberties (CNIL).

### 2.3. Laboratory Methods

Virclia Dengue IgM assay (Vircell, Granada, Spain) is a single-sample chemiluminescence test, including all necessary reagents and controls in a strip. Reaction wells are coated with inactivated dengue antigen Types 1–4. The assay was run on the Virclia Lotus instrument. The turnaround time is estimated at 90 min. Signal values are automatically transformed to antibody index values by the instrument. According to the manufacturer's instructions, samples with an index value below 0, 9, between 0, 9 and 1, 1, and above 1, 1 were interpreted as negative, intermediate, and positive, respectively.

IgM testing was performed at the FRCA using either an “in-house” assay or the commercial Euroimmun Dengue IgM assay. Both tests are microplate-based ELISA methods.

For the in-house assay developed by the FRCA, samples with ratio values below 2.5, between 2.5 and 3, and above 3 were interpreted as negative, intermediate, and positive, respectively. Regarding the Euroimmun IgM assay, the gray zone includes ratio values between 0.8 and 1.1, according to the manufacturer's instructions.

Nucleic acid detection and typing of DENVs were performed at FRCA using a one-step RT-PCR as, previously described [15].

### 2.4. Statistical Analysis

Quantitative data were presented as median and interquartile, and categorical data as percentages. For comparison between assays, intermediate results were considered as positive. Correlation was assessed using Cohen's kappa coefficient (*κ*), and values of 0.81–1.00, 0.61–0.80, 0.41–0.60, and ≤ 0.40 were quoted as excellent, substantial, moderate, and poor correlation, respectively.

## 3. Results

### 3.1. Patients and Dengue Diagnosis

A total of 104 serum samples from 104 patients were included in this study. The characteristics are presented in Table 1. In brief, the median age was 34.3 years old, with a balanced distribution between men and women. They were mostly returning from a trip to the Caribbean (39.4%) and Africa (24.0%). The most common clinical features on admission included fever (88.2%), headache (68.6%), and muscle pain (64.7%). The median time from symptom (TSS) onset to first sample was 6.0 (4–8) days.

Dengue RT-PCR was positive in 57 samples (54.8% of samples). DENV-2 was the most common serotype (49.1%). The typing was undetermined in a significant number of cases (28.1%), and others were DENV-3 (17.5%) and DENV-1 (5.3%).

Most of the positive cases were people returning from a trip to the Caribbean (63.2%) and sampled within 7 days after symptom onset (77.2%).

All cases but one presented as mild dengue fever. The only one case of severe dengue fever was associated with a DENV-2 serotype in a patient who came back from the Caribbean and presented with major asthenia, fever around 40°C, diffuse pain, profuse gingival bleeding, hemoptysis, and gastrointestinal bleeding (hematemesis and rectal bleeding).

### 3.2. Performance of Virclia Dengue IgM Assay for the Diagnosis of Dengue Fever

Dengue IgM assay and dengue RT-PCR results from the 104 samples were plotted together (see Table 2). The overall concordance was 79.8% (83/104), corresponding to a moderate agreement between both assays (kappa = 0.60). When samples were grouped based on TSS, the agreement was 53.3% (16/30) (*k* = 0.07) and 90.5% (67/74) (*κ* = 0.81) in samples with TSS between 0 and 4 days and from Day 5, respectively.

A total of 21 samples were discordant between both assays. The 14 RT-PCR-positive and IgM-negative samples were considered as early collected samples, with a median TSS of 3 days.

The 7 RT-PCR-negative and IgM-positive samples, were resolved using EIA Dengue IgM result, according to the diagnostic algorithm. EIA Dengue IgM was positive in 4 samples considered as true positive, late collected (median TSS of 7 days). EIA result was negative in other samples, which were then considered as negative.

To assess the performance of Virclia IgM assay in diagnosis of dengue fever, RT-PCR positive samples and the samples tested positive by two different IgM assays were considered as true positives.

**Figure 2:**
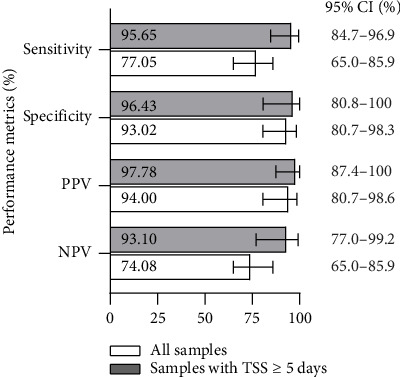
Performance of Virclia Dengue IgM assay for the diagnosis of dengue infection. PPV: positive predictive value; NPV: negative predictive value; TSS: time since symptom; CI: confidence interval.

The sensitivity, specificity, positive predictive value (PPV), and negative predictive value (NPV) of Virclia IgM assay in all samples and in samples with a TSS ≥ 5 days are summarized in Figure 2. In brief, Virclia Dengue IgM testing in samples collected from Day 5 postsymptom onset showed a very good performance in the diagnosis of dengue infection, with sensitivity, specificity, PPV, and NPV estimated at 95.7%, 96.4%, 97.8%, and 93.1%, respectively.

### 3.3. Comparison Between Virclia Dengue IgM Assay and EIA Dengue IgM Assay

A total of 84 samples tested with Virclia Dengue IgM assay were also tested with EIA (in-house or Euroimmun). We took advantage of this subset of samples to investigate the correlation between these 2 types of serological assays.

As shown in Table 3, the overall concordance was 86.9% (73/84), corresponding to a substantial agreement between both assays (kappa = 0.74).

The analysis was then performed separately for samples tested either with in-house EIA (*n* = 29) or Euroimmun EIA (*n* = 55). For in-house EIA, the concordance was 75.9% (22/29), corresponding to a moderate agreement with Virclia assay (kappa = 0.53). Regarding Euroimmun EIA, the concordance was 92.7% (51/55), corresponding to an excellent agreement with Virclia assay (kappa = 0.85).

The 11 discordant results between EIA and Virclia included 7 samples tested by in-house EIA and 4 by Euroimmun EIA. Among the discordant samples, 8 were resolved using RT-PCR results. Dengue RT-PCR was positive in these 8 samples considered as true positive (7 in favor of Virclia and 1 in favor of EIA), early collected samples (median TSS of 5 days). No evidence of serotype impact was observed in discordant samples (2 DENV-1, 3 DENV-2, and 3 DENV-NT). The RT-PCR was negative in the 3 other samples, including two samples tested negative in EIA IgM negative and positive in Virclia IgM, and a third sample that tested EIA IgM positive and Virclia negative. An alternative diagnosis was found in two patients (gonococcal polyarthritis and *Enterovirus* infection).

Finally, during the study period, the accuracy of Virclia Dengue IgM assay was assessed through an External Quality Assessment (EQA) program (Labquality, Helsinki, Finland). A total of 27 samples, including 6 IgM-positive samples and 21 IgM-negative samples. The Virclia IgM results were fully consistent with expected ones.

## 4. Discussion

In Europe, most dengue cases are travelers infected outside of mainland Europe, as shown in our cohort. However, limited outbreaks due to local transmission are increasingly reported, in continental Europe, in areas infested by the rapidly spreading mosquito *Aedes albopictus* [5].

Laboratory confirmation is needed for diagnosis, and detection of viral nucleic acid is considered as the gold standard. Serological assays, especially IgM testing, are also included in guidelines as an important tool for diagnosis, allowing to broaden the time window of diagnosis after symptom onset [8].

In routine clinical practice, IgM testing is usually the first approach implemented, followed, or combined with RT-PCR (dual-testing strategy). This is especially true if IgM testing is performed on an automated platform with results rapidly available, providing useful first information.

We prospectively assessed in this report the performance of the Virclia Dengue IgM assay in clinical samples from patients with suspicion of dengue infection.

Virclia Dengue IgM assay showed a good performance in the diagnosis of dengue infection, especially in samples collected from Day 5 postsymptom onset, with sensitivity and specificity beyond 95%, and an excellent agreement (*k* = 0.81) with RT-PCR result.

These findings are in line with current French guidelines, which recommend nucleic acid testing up to 7 days postsymptom onset and IgM testing from Day 5 [6]. The median TSS in our cohort was 6 days, which is a good reflection of clinical practice, with the need to request both RT-PCR and serology testing from the first medical visit.

Serology can be performed in most clinical laboratories, while samples for RT-PCR are usually sent to a few regional reference centers, with an additional delay to get the results.

Our results also showed an excellent agreement (*k* = 0.85) with the Euroimmun Dengue IgM assay, a conventional plate-based EIA. The Euroimmun Dengue IgM assay has been shown by FRCA to be more sensitive than the former in-house test (unpublished data) and is currently the one in routine use at the FRCA.

Automated platforms allowing single-sample testing are well-adapted for clinical laboratories in a nonendemic area such as metropolitan France. The monotest format with ready-to-use reagents offers flexibility and saves from accumulation of samples for batch testing and reagent loss due to multiple controls. Automation also enhances the quality and efficiency of testing through standardization and reduces turnaround time [16].

To the best of our knowledge, besides the Virclia instrument, commercial and automated dengue IgM testing is only possible on the VIDAS platform. The VIDAS Dengue IgM assay was previously compared to the Panbio Dengue IgM ELISA, with an agreement of 72.5% [12]. In a larger multicentric cohort, the agreement with the same comparator was 82.7% in samples collected at the acute stage [13].

Serological testing is available on the Virclia platform for other arboviruses such as chikungunya and zika, and other agents of febrile syndrome, allowing simultaneous investigation of these pathogens [17]. Nevertheless, nucleic acid testing remains the gold standard for dengue diagnosis, and its implementation on rapid platforms will likely make serology less useful [18, 19].

This study has some limitations. DENV could not be typed in a significant number of samples; however, serotype does not impact IgM detection. In addition, cross-reactions with other flavivirus were not assessed, but the good correlation with RT-PCR is quite reassuring at this point.

In conclusion, the Virclia Dengue IgM assay showed a good performance in the diagnosis of dengue infection and can be used in combination with RT-PCR for laboratory confirmation. The automation coupled with the monotest format is well-adapted for nonendemic areas.

## Figures and Tables

**Table 1:** Patients' characteristics on admission.

Characteristics (*n* = 104)	
Median age, years (IQR)	34.3 (25.3–51.5)

*Sex (%) (n = 104)*	
Male	49.0
Female	51.0

*Traveling from (%) (n = 104)*	
The Caribbean	39.4
Africa	24.0
Asia/Oceania	17.3
Latin America	14.4
Other	4.8

*Clinical features (%) (n = 104)*	
Fever	88.2
Headache	68.6
Muscle pain	64.7
Gastrointestinal symptoms	33.3
Joint pain	32.4
Rash	25.5
Retro-orbital pain	14.7
Hemorrhagic signs	10.8

*Time since symptom (%) (n = 104)*	
≤ 4 days	28.8
5–7 days	41.3
> 7 days	29.8

**Table 2:** Agreement between Virclia Dengue IgM assay and Dengue RT-PCR.

Samples	Virclia Dengue IgM assay	Dengue RT-PCR			Agreement (kappa)
		Positive	Negative	Total	
All samples	Positive	43	7	50	79.8% (0.60)
	Negative	14	40	54	
	Total	57	47	104	

Samples with TSS 0–4 days	Positive	3	2	5	53.3% (0.07)
	Negative	12	13	25	
	Total	15	15	30	

Samples with TSS ≥ 5 days	Positive	40	5	45	90.5% (0.81)
	Negative	2	27	29	
	Total	42	32	74	

**Table 3:** Agreement between Virclia Dengue IgM assay and ELISA Dengue IgM assay.

Samples	Virclia Dengue IgM assay	ELISA dengue IgM assay			Agreement (kappa)
		Positive	Negative	Total	
All samples	Positive	33	9	42	86.9% (0.74)
	Negative	2	40	42	
	Total	35	49	84	

Samples tested with in-house assay	Positive	12	6	18	75.9% (0.53)
	Negative	1	10	11	
	Total	13	16	29	

Samples tested with Euroimmun assay	Positive	21	3	24	92.7% (0.85)
	Negative	1	30	31	
	Total	22	33	55	

## Data Availability

The data that support the findings of this study are available on request from the corresponding author. The data are not publicly available due to privacy or ethical restrictions.
